# Role of Quantum Entropy and Establishment of H-Theorems in the Presence of Graviton Sinks for Manifestly-Covariant Quantum Gravity

**DOI:** 10.3390/e21040418

**Published:** 2019-04-19

**Authors:** Massimo Tessarotto, Claudio Cremaschini

**Affiliations:** 1 Department of Mathematics and Geosciences, University of Trieste, Via Valerio 12, 34127 Trieste, Italy; 2 Institute of Physics, Faculty of Philosophy and Science, Silesian University in Opava, Bezručovo nám.13, CZ-74601 Opava, Czech Republic; 3 Institute of Physics and Research Center for Theoretical Physics and Astrophysics, Faculty of Philosophy and Science, Silesian University in Opava, Bezručovo nám.13, CZ-74601 Opava, Czech Republic

**Keywords:** covariant quantum gravity, Gaussian solutions, Boltzmann–Shannon entropy, H-theorem, 03.65.Ca, 03.65.Ta, 04.60.-m, 04.70.Dy

## Abstract

Based on the introduction of a suitable quantum functional, identified here with the Boltzmann–Shannon entropy, entropic properties of the quantum gravitational field are investigated in the framework of manifestly-covariant quantum gravity theory. In particular, focus is given to gravitational quantum states in a background de Sitter space-time, with the addition of possible quantum non-unitarity effects modeled in terms of an effective quantum graviton sink localized near the de Sitter event horizon. The theory of manifestly-covariant quantum gravity developed accordingly is shown to retain its emergent-gravity features, which are recovered when the generalized-Lagrangian-path formalism is adopted, yielding a stochastic trajectory-based representation of the quantum wave equation. This permits the analytic determination of the quantum probability density function associated with the quantum gravity state, represented in terms of a generally dynamically-evolving shifted Gaussian function. As an application, the study of the entropic properties of quantum gravity is developed and the conditions for the existence of a local H-theorem or, alternatively, of a constant H-theorem are established.

## 1. Introduction

The understanding of the abstract, i.e., geometrical, structure of space-time poses challenging mathematical questions that have to be established on rigorous grounds. In this context, fundamental issues concern in particular the role and precise identification of the entropic properties of space-time (i.e., the “universe”). This paper has the goal of establishing a theoretical background for the notion of quantum entropy in the context of quantum gravity (QG) theory, to be identified with a four-scalar quantum physical observable. We intend to show in particular that such a quantum observable is actually local, in the sense that it can be associated with arbitrary physical locations $r\equiv \left\{r^{\mu }\right\}$ of an assigned background space-time. The notion of quantum gravity entropy proposed here has a wide applicability, and hence, it is not related to either a non-local or a locally-defined quantity corresponding exclusively to black holes (BH) and/or related event horizons (EH). Nevertheless, since classical black hole environments are expected to play a relevant role in the entropic aspects of quantum gravity, as an application of the theory, the particular case of the de Sitter EH cosmological solution is considered, which allows also for the inclusion of possible spatially-localized quantum loss effects (*localized quantum sinks or pits*; see also the related discussion in Section 2).

A preliminary basic prerequisite is the identification of an appropriate realization, either discrete or continuous, of the theory of quantum gravity (QG), i.e., a quantization scheme for the theory of the gravitational field, which is realized by the standard formulation of GR (SF-GR), namely Einstein’s gravitational field equations. Taking into account the theoretical premises set in [1,2], such a theory should be able at a minimum:

- *Prescription #1*: To prescribe a quantum-wave equation for the relevant quantum state (ψ) associated with the corresponding quantum particle, i.e., the graviton.
- *Prescription #2*: To determine also the corresponding quantum hydrodynamics equations, i.e., the quantum continuity equation for the probability density function (PDF) and the quantum Hamilton– Jacobi equation for the related quantum phase-function.
- *Prescription #3*: To prescribe a suitable set of quantum observables and corresponding “macroscopic” physical variables expressed as quantum expectation values of the former ones, both identified with four-tensors defined with respect to a background space-time structure to be determined.
- *Prescription #4*: To determine also the same geometric background structure of the universe, to be identified with a four-dimensional differential manifold $\left\{Q^{4},\hat{g}\right\}$ defined in terms of an appropriate “background” metric tensor, which is represented equivalently in terms of its co- and counter-variant tensor representation:
  (1) $$\hat{g}\equiv \left\{{\hat{g}}_{\mu \nu }\right\}\equiv \left\{{\hat{g}}^{\mu \nu }\right\},$$
  and raises and lowers the tensor indices of the remaining tensor fields of the theory, where lower and upper indices are denoted respectively as covariant and countervariant. Hence, an arbitrary tensor field, which in its covariant representation is given by $A_{\mu \nu }$, in its countervariant representation is just:
  (2) $$A^{\mu \nu }={\hat{g}}^{\mu \alpha }{\hat{g}}^{\nu \beta }A_{\alpha \beta }.$$
  Specifically, consistent with the so-called emergent-gravity picture, the latter should be a macroscopic variable and thus coincide with the quantum expectation value of a corresponding appropriate quantum (i.e., intrinsically stochastic) observable.
- *Prescription #5*: To prescribe, consistent with the quantum-wave equation, also the set of corresponding partial differential equations that determines the same background metric tensor, to be referred to as the tensor quantum-modified Einstein’s field equation (see the related discussion in [3]).
- *Prescription #6*: The background metric tensor is required to be a non-stationary tensor field, i.e., of the general form:
  (3) $$\hat{g}\left(r,s\right)\equiv \left\{{\hat{g}}_{\mu \nu }\left(r,s\right)\right\},$$
  which depends both on the four-position $r\equiv \left\{r^{\mu }\right\}$ and the proper time *s*. More precisely, here, *s* denotes the dynamical time-parameter of the theory, which is identified with a suitably-prescribed invariant proper-time parameter associated with subluminal geodesic trajectories of the same background metric tensor. Furthermore, by assumption, $\hat{g}\equiv \left\{{\hat{g}}_{\mu \nu }\left(r,s\right)\right\}$ identifies a particular solution of the tensor quantum-modified Einstein field equation.
- *Prescription #7*: To be consistent with quantum mechanics, quantum field theory, and simultaneously also with the principles of SF-GR, with particular reference to the principles of general and manifest covariance.

Concerning in particular the notion of proper-time introduced in *Prescription #6*, we refer here to the extended discussion reported in [3]. Accordingly, proper time can have two physical interpretations. In the first one, *s* is the observer proper-time, i.e., a local observable that may have nevertheless different realizations for each observer (i.e., GR-frames that are mutually connected via local coordinate transformations). In this case, the proper-time *s* is by construction the same one for all geodesic trajectories that cross simultaneously the generic observer four-position. The second possible realization is provided instead by the notion of global proper-time, i.e., a global observable that is the same one also for a family of observers that are properly “synchronized” with each other in such a way that the observer proper-time *s* indeed coincides for all of them. In this case, the observer proper-time *s* takes therefore, by suitable construction, the same value for all such observers.

Provided a quantum theory satisfying the previous prescriptions exists that preserves also the dimension of space-time, the space-time structure indicated above should be associated with a continuum four-dimensional Riemannian differential manifold $\left\{Q^{4},\hat{g}\right\}$. In addition, $\hat{g}$ and the same equations should be determined without resorting to the semiclassical limit and making use only of the same quantum state ψ and its corresponding quantum-wave equation.

Despite different possible expectations, it is obvious that the quest for a unique and fully-consistent formulation of QG remains still (partially) unsolved to date due to the formidable difficulty of the problem set above. Nevertheless, a mandatory requirement for its determination is certainly the principle of manifest covariance (PMC), which thanks to the previous *Prescription #7* is set both at the basis of SF-GR and QG alike. In fact, the same principle demands the invariance property of the theory with respect to the group of local point transformations (LPT) associated with the same differential manifold $\left\{Q^{4},\hat{g}\right\}$. The latter is identified with the ensemble of diffeomorphism, i.e., continuously differentiable, LPT of the form:

(4) $$r^{\mu }\to r^{\prime }{}^{\mu }=r^{\prime }{}^{\mu }\left(r\right),$$

mapping in each other coordinate system (or GR-frames), $r\equiv \left\{r^{\mu }\right\}$ and $r^{\prime }\equiv \left\{r^{\prime }{}^{\mu }\right\}$. Thus, it follows that $\left\{Q^{4},\hat{g}\left(r\right)\right\}\equiv \left\{Q^{4},{\hat{g}}^{\prime }\left(r^{\prime }\right)\right\}$, with *r* and $r^{\prime }$ possibly identifying also different admissible physical “locations” on the same space-time.

A number of mandatory physical consequences follow.

The first one is that for QG, only continuous representations of the background space-time actually appear able to provide a viable solution that is strictly compatible with PMC (thus, on the contrary, discrete models of QG should be ruled out at this stage). In fact, the same background structure of space-time appears simultaneously: (a) continuous, i.e., such that all physical laws can be expressed locally for arbitrary GR-frames; (b) homogenous, i.e., such that all physical laws must be expressed in four-tensor form with respect to the LPT-group; (c) frame-independent, i.e., the same laws take the same form everywhere in the whole universe.

That such a background space-time structure of a similar type actually applies to QG also follows from the discovery of the classical and reduced-dimensional Hamiltonian structures of SF-GR represented by the covariant classical theory of SF-GR (CCG-theory) reported in [1]. The basic characteristic of the manifestly-covariant Hamiltonian representation determined in this way is that it is constraint-free, a feature that makes possible the adoption of a manifestly-covariant standard canonical quantization method and the development of a corresponding manifestly-covariant formulation of QG (referred to as CQG-theory; see [1,2]). This includes in particular the construction of corresponding manifestly-covariant quantum wave equation (the CQG-wave equation, [2]) providing an evolution equation for the four-scalar quantum state. We refer in particular to the subsequent Section 2 for further motivations and basic implications.

That the property of manifest covariance and the very notion of background space-time are intrinsically built in the same quantum theory emerges, in fact, also from the fact that the quantum-modified Einstein field equations follow self-consistently as indicated above (i.e., without performing a semiclassical limit) from the same quantum wave equation (see [3]).

Therefore, we conclude that a crucial aspect of CQG-theory, as well as SF-GR and QG alike, is indeed played by the principle of manifest covariance (see *Prescription #7*). Indeed, CQG-theory is characterized by the fact that all the dynamical variables and quantum operators that characterize the theory are expressed in terms of continuum field variables that behave as four-tensors with respect to the LPT-group (see Equation (4)), i.e., the group of point transformations which preserve the differential manifold structure of space-time.

Given these premises, the present paper is part of a research effort about the theoretical foundations and principles of classical and quantum gravity. In particular, this paper deals with new developments of CQG-theory, which concern specifically the solution of two basic conceptual issues:

- The first one is the problem of the “a priori” prescription of the quantum entropy for the quantum gravitational field. In the context of quantum gravity, the notion is to be intended (in analogy with information theory and statistical mechanics) as a local measure of “quantum ignorance”, namely associated with its quantum state. In other words, it should prescribe the ignorance about the knowledge of the four-scalar quantum state $\psi =\psi (\hat{g},g,r,s)$, which in the framework of the same theory is associated with a spin-two graviton particle. These requirements should be realized by identifying the quantum entropy in terms of a suitable quantum expectation value, i.e., a functional of ψ. This feature should warrant at the same time that the same definition, to make sense at all, should be possible not merely only on event horizons (EH) of black holes (BH), but hold also for arbitrary space-time four-positions. In addition, to warrant its frame-independent feature, quantum entropy should necessarily be identified with a real four-scalar, i.e., just as the quantum state ψ an invariant with respect to the LPT-group of local point transformations (LPT).
- The second aspect deals with the quantum treatment of event horizons (EH) and the long-standing question of whether a phenomenon of loss of information may actually occur, in some sense, at the same location, despite the fact that EH represent absolute barriers for classical matter and radiation alike. An equivalent question is whether EH may nonetheless be characterized by the absorption (or emission) of the quanta of the gravitational field, i.e., the gravitons, which even at the EH (may be expected to) exhibit a finite probability density of crossing the same EH. In particular, for definiteness here, the case shall be considered in which $\left\{Q^{4},\hat{g}\right\}$ coincides with the de Sitter space-time and the same event horizon is the corresponding de Sitter EH.

In order to address these questions, in the following, a quantum-non-unitarity generalization of CQG-theory will be carried out and a corresponding formulation of the generalized Lagrangian path approach (GLP-approach) will be subsequently discussed. The crucial feature, which we intend to display in this paper, is that in this way the entropic properties of quantum gravity can be investigated with particular reference to the validity of a local strong H-theorem, or alternatively of constant H-theorems holding for finite proper-times *s*, or in the asymptotic limit s→+∞. This is realized by first identifying the quantum entropy functional with the Boltzmann–Shannon (BS) entropy for the quantum gravity PDF. In particular, it is shown that in the absence of quantum sinks, the BS entropy in a vacuum is locally conserved in the presence of a stationary background space-time, thus warranting the validity of a so-called constant H-theorem. Instead, sufficiently close to the de Sitter event horizon, if localized quantum sinks are present, the BS entropy generally exhibits an irreversible proper-time monotonically-increasing behavior as corresponds to the occurrence of a strictly-positive entropy production rate.

### 1.1. Historical Setting and Background

The concept of entropy emerges in many areas of physics and is customarily related to the finite/ asymptotic time-evolution of dynamical systems occurring both in classical [4,5] and quantum [6,7] theories. Incidentally, this involves also the notion of some suitable effective time on which the dynamics of the same systems should depend and which characterizes their “microscopic” behavior. This may be identified either with the coordinate time (or absolute time in the case of non-relativistic systems) or, more generally, as it should necessarily be in the case of relativistic systems, by a four-scalar proper time to be appropriately prescribed.

Regarding the possible prescription of entropy, a notable example is provided by its identification with the Boltzmann–Shannon (BS) entropy [8]. An instance of this kind is represented by the statistical treatment of *N*-body classical systems, a notable example-case being represented by *N*-body hard-spheres of radius σ or (equivalently) point particles subject to spherically-symmetric binary interactions of finite radius σ. In both cases, this occurs when the so-called Boltzmann–Grad limit is evaluated (according to [9], this is obtained by suitably letting N→∞ and σ→0, while keeping the product $N\sigma^{2}$ constant). In such a case, the BS statistical entropy is usually associated with the property of “macroscopic” irreversibility (i.e., the so-called H-theorem), which characterizes the statistical behavior of these systems [10]. Remarkably, the same property holds, in contrast to the “microscopic” one, despite the fact that the same classical dynamical systems are time-reversal invariant (i.e., time-reversal symmetric [11]). Nevertheless, for more general classical dynamical systems, other definitions are actually appropriate (see again [11]). In the case of classical dynamical systems, this occurrence arises for finite hard-sphere systems, namely in which the parameters (N,σ) remain finite.

Nevertheless, the concepts of entropy and entropy production arise also for quantum systems [12,13]. An example case worth mentioning is represented by the Schrödinger dynamical system, i.e., the dynamical system that is associated with the Schrödinger equation [14]. As shown in [15], the BS entropy associated with the corresponding quantum probability density may exhibit a well-definite asymptotic behavior. In fact, in the case of asymptotic-free quantum particles, as discovered in the same reference, a constant H-theorem holds.

It must be noted, however, that although disparate possible definitions of quantum entropy are in principle available for the treatment of a given physical system, its appropriate choice—arising in different contexts and/or theoretical settings—depends crucially on its physical interpretation. For example, in classical mechanics for continuum systems, one can introduce the notions of thermodynamic entropy, statistical entropy, as well as entropy associated with information theory (e.g., the BS and Fisher entropies) and logical entropy [16,17,18,19,20,21]. Some of these concepts (of entropy) pertain also to continuum classical field theories. For the gravitational field theory described in the framework of general relativity (GR), the background is founded on the seminal works by Hawking and Beckenstein and the black hole surface-area entropy relation [22,23,24,25,26] given by them. This result has supported the conjecture that space-time possesses a discrete microscopic structure that could be described by means of a quantum theory of gravity and that classical GR should emerge as its macroscopic continuum (i.e., thermodynamic) limit [27,28,29,30]. The study of entropic properties of black holes and black hole event horizons has gained an increasing interest together with the role of quantum corrections in this context [31,32,33]. Nevertheless, even for the treatment of the gravitational field in vacuum conditions, one should be able to introduce a general notion of entropy, and in particular quantum entropy, which applies to the gravitational field itself as a result of its very nature and in particular its possible dynamical evolution. Such a definition should be independent of the possible existence of classical black holes and related processes and should therefore be of general applicability, without involving necessarily notions that are specific to black hole structures. In fact, although the notion of thermodynamic entropy for the gravitational field is rooted in classical GR and classical black hole theory, its principles can be extended to include currently also the case of quantum gravity (QG; see for example [34,35,36,37,38,39]). The same conjecture is expected to apply to the BS statistical entropy, which originates specifically from classical statistical mechanics (CSM) and information theory and for this reason appears more suitable to extend its meaning to the probabilistic setting characteristic of a quantum theory. In the case of the gravitational field, an entropic theory of this type should satisfy the following basic principles:

(1) To preserve the validity of the covariance and manifest covariance that characterize classical GR and a quantum theory of the gravitational field that should stem from it. This criterion is consistent with the quantum objectivity principle of quantum physics (see for example [40,41]).
(2) To preserve the validity of the conceptual framework of quantum field theory, including the probabilistic physical interpretation of the wave function, the definitions of quantum operators, and observables (see again [40,41]).
(3) To preserve the connection with information theory regarding the physical interpretation of the BS statistical entropy as a measure of the ignorance of the information available on the physical state of a system (see [8,16,17]).
(4) To admit, in analogy to quantum mechanics, a definition that applies both to stationary and non-stationary quantum states, provided the same dynamical evolution is expressed in terms of a manifestly-covariant parameter.

### 1.2. Goals

In this paper, progress in the theory of QG is reported that may actually help in achieving insight into the possible role of quantum gravity entropy. In this context, the latter is expected to be identified, in analogy to CSM, with the BS statistical entropy associated with the relevant quantum probability density function (PDF) characteristic of the theory. Based on such a statistical definition of entropy, it is conjectured that an entropy generation rate should generally correspond to the occurrence of non-stationary (in some suitable sense) quantum gravity states, while the vanishing of the same entropy rate should identify appropriate equilibrium/stationary quantum states. More precisely, to this end, reference will be made to recent developments of QG that concern the adoption of manifestly-covariant canonical approaches for the quantization of the space-time. This means, in particular, that both the quantum PDF and the same quantum entropy must be prescribed in terms of suitable four-scalars, with the second one being associated with an appropriate quantum expectation value of the quantum PDF itself.

Emphasis is devoted in particular to a number of unsolved related issues, which include:

- The definition of statistical entropy in QG and the consequent extension of the notion of BS entropy to the treatment of quantum gravity states.
- The explicit construction of the quantum entropy in vacuum together with its corresponding entropy production rate and the understanding of the physical mechanisms for its occurrence.
- The investigation of the asymptotic behavior of the quantum entropy and, correspondingly, the possible validity, in analogy to non-relativistic quantum mechanics, of an asymptotic and/or local constant H-theorem.
- The role of the cosmological constant in the determination of the quantum entropy and the related issue of its physical origin.

In order to address these issues, in this paper, the theoretical framework represented by the theory of manifestly-covariant quantum gravity (*CQG-theory*) is adopted (see [1,2,3,42,43,44,45]). This provides a self-consistent theory of quantum gravity for the standard formulation of general relativity, which satisfies simultaneously all the physical *Prescriptions #1–#7* set above, including quantum mechanics, quantum field theory [46], classical Einstein theory of GR [47,48,49], and in particular the principles of covariance and manifest covariance [50,51].

The scheme of the paper is as follows. In Section 2, a review of the fundamental concepts of manifestly-covariant quantum gravity theory is given, together with the necessary non-unitary generalization concepts. Section 3 contains a summary of the GLP formalism and the corresponding GLP formulation of non-unitary CQG-theory. In Section 4, the definition of the CQG Boltzmann–Shannon entropy and the corresponding entropy production rate are introduced. Entropic properties of the analytic quantum solution and the search of H-theorems for the quantum gravitational field are discussed in Section 5. Concluding remarks are reported in Section 6, while additional notations for the classical and quantum Hamiltonian structures of GR are reported for completeness in the Appendix A.

## 2. Covariant Quantum Gravity Theory: Domain Validity and Non-Unitary Generalization

The formulation of the manifestly-covariant quantum theory of the gravitational field, i.e., CQG-theory, actually pertains to different possible subsets of space-time. Here in particular, as in [3], the general case is considered of a deterministic non-stationary background metric tensor of the type (3), which includes both implicit and explicit proper-time *s*-dependences. However, for the prescription of *s*, different subsets of space-time must be distinguished. In fact, besides the possible presence of an “external” de Sitter event horizon, the existence of a multitude of local black holes (BH) and event horizons (EH) needs to be accounted for. Thus, referring in particular to the domain around a given isolated BH, this means that, denoting by $dr^{\mu }$ an arbitrary infinitesimal four-displacement along a geodesic curve r=r(s) belonging to the four-position $r\equiv \left\{r^{\mu }\right\}$, the following subsets of space-time can be distinguished:

(5) $${\hat{g}}_{\mu \nu }\left(r,s\right)dr^{\mu }dr^{\nu }=\left\{\begin{array}{c} >0\;(\mathrm{outer-EH}\;\mathrm{domain}),\;\\ =0\;(\mathrm{EH}),\;\\ <0\;(\mathrm{inner-EH}\;\mathrm{domain}). \end{array}\right.$$

In particular, the subsets indicated above identify respectively the outer, the EH itself, and the inner domains with respect to the same EH. The validity of the functional dependence (3) for $\hat{g}$ and its consistency with the classical GR setting was established in [3] in the case of outer-EH domains. This implies in particular that the invariant proper-time parameter *s* was identified in such a case with the Riemann distance evaluated along two four-positions $r\equiv \left\{r^{\mu }\right\}$ and $r_{A}\equiv \left\{r_{A}^{\mu }\right\},$ which belong to the same subset, i.e., the outer-EH domain, and are connected by a geodesic curve $C_{\left(r,r_{A}\right)}$, namely:

(6) $$s=s_{A}+\int_{C_{\left(r,r_{A}\right)}}\sqrt{{\hat{g}}_{\mu \nu }\left(r,s\right)dr^{\mu }dr^{\nu }}.$$

However, corresponding to the domain classification (5), a more general prescription is possible. In fact, the infinitesimal Riemann distance ds along the same geodesic can be respectively identified with:

(7) $$ds^{2}=\left\{\begin{array}{c} {\hat{g}}_{\mu \nu }\left(r,s\right)dr^{\mu }dr^{\nu }\;>0\;(\mathrm{outer-EH}\;\mathrm{domain}),\;\\ {\hat{g}}_{\mu \nu }\left(r,s\right)dr^{\mu }dr^{\nu }\equiv 0\;(\mathrm{EH}),\;\\ -{\hat{g}}_{\mu \nu }\left(r,s\right)dr^{\mu }dr^{\nu }\;>0\;(\mathrm{inner-EH}\;\mathrm{domain}). \end{array}\right.$$

Hence, both in the inner-EH and outer-EH domains (as well as on the same EH), it follows that the prescription:

(8) $$ds^{2}=\left|{\hat{g}}_{\mu \nu }\left(r,s\right)dr^{\mu }dr^{\nu }\right|$$

necessarily holds. As a consequence, the parameter *s* identifies in both cases the finite Riemann distance between two different four-positions $r\equiv \left\{r^{\mu }\right\}$ and $r_{A}\equiv \left\{r_{A}^{\mu }\right\},$ which are connected by a geodesic curve $C_{(r,r_{A})\;}$ and belong to the same subset, namely:

(9) $$s=s_{A}+\int_{C_{\left(r,r_{A}\right)}}\sqrt{\left|{\hat{g}}_{\mu \nu }\left(r,s\right)dr^{\mu }dr^{\nu }\right|},$$

while:

(10) $$s=s_{A}\equiv const.$$

identically on all event horizons. The conclusion is therefore that with such a prescription, the domain validity of CQG-theory actually extends to arbitrary subsets of space-time according to the classification given above (i.e., Equation (5)). This means in particular that a first-quantization quantum theory of gravity satisfying the principle of unitarity, i.e., conservation of quantum probability, is warranted in all cases.

However, the question arises whether in certain subsets of space-time, this property may be violated. The issue is in fact whether a non-unitary generalization of CQG-theory is actually achievable. The physical motivation is the possible occurrence of second-quantization effects leading to the creation/destruction of gravitons, and in particular the possible presence of an appropriate subset of EH of spatially-localized quantum sinks (or capture terms) in the CQG-wave equation. Event horizons of this type are expected to include in particular the de Sitter EH, namely the EH that characterizes the de Sitter cosmological solution of the quantum-modified Einstein field equations found in [3].

The conjecture is that these effects might arise in suitable subsets of space-time occurring sufficiently close to the EH allowing, in particular, gravitons to decay or effectively to cross an EH. The result is well-known in quantum mechanics, leading to a violation of quantum unitarity. This should correspond to the appearance of an effective capture mechanism that determines a corresponding decrease of the quantum probability density of the gravitons sufficiently close to the same EH. Accordingly, given a spherical (or spherical-like) coordinate system, the phenomenon should occur if the radial separation of a graviton particle from the EH $\left|r_{c}-r_{o}\right|$ (with $r_{c}$ and $r_{o}$ being respectively the radial coordinates of the particle and of the EH) is suitably small. Thus, if one introduces the dimensionless parameter:

(11) $$\Delta \equiv \frac{\left|r_{c}-r_{o}\right|}{r_{o}},$$

one can distinguish the two subsets of space-time for which respectively:

(12) $$\Delta =\left\{\begin{array}{c} \gg \varepsilon \;(\mathrm{EH}\;\mathrm{non-capture}\;\mathrm{domain}),\;\\ ∼\varepsilon \;(\mathrm{EH-capture}\;\mathrm{domain}), \end{array}\right.$$

with the two domains being referred to respectively as EH non-capture and capture-domains, and ε≪1 is a small parameter. For definiteness, in the following, we shall also assume that the region close to the EH identified by the EH-capture domain is characterized by the presence of second-quantization effects arising in the CQG quantum-wave equation and due alternatively to the occurrence of a quantum sink (or capture) or respectively of a quantum source term. Depending on the subset that is considered, in particular whether it coincides with the outer-EH or inner-EH domain, this effect may correspond to the possible effective presence of a quantum sink, i.e., a capture mechanism (respectively a source, i.e., source mechanism) implying the possible local violation of the quantum unitarity principle.

More precisely, regarding the possible physical mechanism leading to the propagation of gravitons beyond the EH, the conjecture is as follows. When the graviton is approaching the EH coming from the outer domain, one notices that in the EH-capture domain, the physical propagation condition ${\hat{g}}_{\mu \nu }\left(r,s\right)dr^{\mu }dr^{\nu }\geq 0$ should be actually replaced by the stochastic quantum condition:

(13) $$\Delta g_{\mu \nu }dr^{\mu }dr^{\nu }\geq 0,$$

where $\Delta g_{\mu \nu }$ is the stochastic quantum displacement field introduced in Section 3, prescribed so that its normalized quantum expectation value (see Equation (74) below) coincides with the background metric tensor ${\hat{g}}_{\mu \nu }$. In fact, according to the GLP formalism described below, $\Delta g_{\mu \nu }$ is simply one of the infinite admissible stochastic values of the quantum metric tensor that graviton particles can carry, and therefore, it is an intrinsic property of the graviton. As a consequence, provided the inequality (13) is satisfied, the same graviton has a finite probability density of propagating through the EH. In principle, this behavior may be expected to occur both in the outer and inner regions of a given EH, corresponding respectively to outgoing and incoming gravitons. However, there may exist EH in which the predominant physical effect is due to outgoing gravitons, namely that escape across the same EH. This effectively corresponds to a sink or loss mechanism for graviton quantum particles.

An example of EH satisfying these conditions is the one occurring in the cosmological de Sitter space-time, which as shown in [3], is a solution of the tensor quantum-modified Einstein field equation in a vacuum with a non-vanishing cosmological constant. Such a solution is peculiar for several reasons:

- First, because it is characterized by a proper-time-dependent cosmological constant Λ=Λ(s). The initial condition of the same cosmological constant is set conventionally at proper time s=0, i.e., the Big Bang event, so that $\Lambda_{o}=\Lambda \left(s=0\right)$.
- Second, the space-time subset in which the solution is defined is bounded. Its boundary coincides with an EH that identifies the de Sitter EH.
- Third, on the de Sitter EH, the cosmological constant remains time-independent. Therefore, its value must coincide with the Big Bang initial condition for the same cosmological constant, i.e., $\Lambda_{o}$.
- Fourth, because the de Sitter EH is an external boundary of space-time, this means that it is an ideal candidate for the possible existence of an EH-capture domain for gravitons, namely characterized by a quantum loss mechanism of graviton particles. Nevertheless, this does not rule out the possibility of having additional classical or quantum sources of matter or radiation (such as the Hawking radiation), not involving however the creation or destruction of graviton particles.

Specifically, the goal is to investigate, whether and under what conditions, in such a case the connections with the quantum hydrodynamic equations (cf. *Prescription #2* above), as well with the quantum-modified Einstein field equations (cf. *Prescription #5*), previously pointed out in the framework of CQG-theory in [3,45], both still apply.

The starting point is the prescription of the quantum-wave equation, which in the two domains advances the quantum-wave function $\psi \left(g,s\right)\equiv \psi \left(g,\hat{g},r,s\right)$, with r=r(s) denoting for arbitrary *s* belonging to the time axis I≡R in principle along an arbitrary geodesic curve of $\left\{Q^{4},\hat{g}\right\}$. As in [2], this is based on the canonical quantization scheme of the classical Hamiltonian structure of GR, which is associated with the Einstein field equations, i.e., *Hamilton–Jacobi g*-*quantization*, namely through the mapping:

(14) $$\begin{array}{c} \left.g_{\mu \nu }\to g_{\mu \nu }^{\left(q\right)}\equiv g_{\mu \nu },\right. \end{array}$$

(15) $$\begin{array}{c} \left.\pi_{\mu \nu }\equiv \frac{\partial S(g,\hat{g},r,s;P)}{\partial g^{\mu \nu }}\to \pi_{\mu \nu }^{\left(q\right)}\equiv -i\hbar \frac{\partial }{\partial g^{\mu \nu }},\right. \end{array}$$

(16) $$\begin{array}{c} \left.p\equiv -\frac{\partial S(g,\hat{g},r,s;P)}{\partial s}\to p^{\left(q\right)}\equiv -i\hbar \frac{d}{ds},\right. \end{array}$$

with $g_{\mu \nu },$ $\pi_{\mu \nu }\equiv \frac{\partial S(g,\hat{g},r,s;P)}{\partial g^{\mu \nu }}$ and $p\equiv -\frac{\partial S(g,\hat{g},r,s;P)}{\partial s}$ denoting the classical Lagrangian coordinates and the corresponding momenta, which are conjugate to $g^{\mu \nu }$ and *s*, respectively, while $g_{\mu \nu }^{\left(q\right)},$ $\pi_{\mu \nu }^{\left(q\right)}$ and $p^{\left(q\right)}$ are the quantum operators. However, departing from [2], the mapping for the classical Hamiltonian function $H_{R}\left(g,\frac{\partial S(g,\hat{g},r,s;P)}{\partial g},\hat{g}\left(s\right),r,s\right)$ (which is recalled in Appendix A) is taken now as the more general form:

(17) $$H_{R}\left(g,\frac{\partial S(g,\hat{g},r,s;P)}{\partial g},\hat{g}\left(s\right),r,s\right)\to H_{R}^{\left(q\right)}+\frac{i\hbar }{2}Q_{L}^{\left(q\right)},$$

where $H_{R}^{\left(q\right)}$ identifies the corresponding quantum Hamiltonian operators and $i\hbar Q_{L}^{\left(q\right)}$ and $Q_{L}^{\left(q\right)}$ denote respectively an appropriate four-scalar sink (or capture) quantum operator and a real function, denoted as a graviton capture term. In particular, $Q_{L}^{\left(q\right)}$ will be assumed coordinate-independent and therefore of the form:

(18) $$Q_{L}^{\left(q\right)}=f\left(r,s\right)\Theta \left(\varepsilon -\Delta \right),$$

with f(r,s) being an arbitrary smooth and bounded real function. As a consequence, $Q_{L}^{\left(q\right)}$ is non-zero only in the EH domain defined above. In particular, in the case in which f(r,s)≤0 (respectively ≥0), the operator $\frac{i\hbar }{2}Q_{L}^{\left(q\right)}$ identifies a localized quantum *sink* or *capture* (respectively a *source*) operator. As a consequence, consistent with such a quantization scheme, it follows that the quantum-wave equation can be identified with the elliptic evolution PDE of the general form:

(19) $$i\hbar \frac{d}{ds}\psi \left(g,s\right)=\left\{H_{R}^{\left(q\right)}+\frac{i\hbar }{2}Q_{L}^{\left(q\right)}\right\}\psi \left(g,s\right).$$

The remaining notations are recalled in Appendix A. Thus, in particular, $\frac{d}{ds}$ denotes the total covariant *s*-derivative (A5), while $H_{R}^{\left(q\right)}$ is the self-adjoint quantum Hamiltonian operator prescribed by Equation (A8). Such an equation is referred to as the *CQG-wave equation.*

The CQG-wave Equation (19) can be represented in terms of an equivalent set of quantum hydrodynamic equations [2,44] by introducing the Madelung representation:

(20) $$\psi \left(g,\hat{g},r,s\right)=\sqrt{\rho (g,\hat{g},r,s)}\exp\left\{\frac{i}{\hbar }S^{\left(q\right)}\left(g,\hat{g},r,s\right)\right\},$$

where the quantum fluid fields $\left\{\rho ,S^{\left(q\right)}\right\}\equiv \left\{\rho \left(g,\hat{g},r,s\right),S^{\left(q\right)}\left(g,\hat{g},r,s\right)\right\}$ identify respectively the four-scalar *effective* quantum PDF and quantum phase-function. Notice in particular that $\rho (g,\hat{g},r,s)$ can be identified with a probability density only in the non-EH domain where by construction:

(21) $$\int_{U_{g}}d\left(g\right)\rho \left(g,\hat{g},r,s\right)=1,$$

with $U_{g}\subseteq R^{10}$ denoting the 10-dimensional configuration space spanned by the symmetric coordinate field $g\equiv \left\{g_{\mu \nu }\right\}$, whereas in the EH domain and in the presence of a quantum sink, it generally follows that:

(22) $$\int_{U_{g}}d\left(g\right)\rho \left(g,\hat{g},r,s\right)\leq 1.$$

In terms of the Madelung representation (20) and in analogy with the formulation of CQG-theory reported in [3,45], the corresponding set of quantum hydrodynamic equations of (CQG-QHE) is therefore recovered. These are now realized by the PDE’s in Eulerian form:

(23) $$\begin{array}{ccc} \frac{d\rho }{ds}+\frac{\partial }{\partial g_{\mu \nu }}\left(\rho V_{\mu \nu }\right) & = & Q_{L}^{\left(q\right)}, \end{array}$$

(24) $$\begin{array}{ccc} \frac{dS^{\left(q\right)}}{ds}+H^{\left(q\right)} & = & 0. \end{array}$$

Notice, in particular, that the first equation realizes the non-unitary generalization of the quantum continuity equation, while the second one remains unchanged with respect to conventional CQG-theory and coincides with the quantum Hamilton–Jacobi equation. Here, the notation is standard. Thus, $\frac{d}{ds}$ coincides again with the convective derivative $D_{s}$ (see Equation (A7) in Appendix A), $V_{\mu \nu }$ and $H^{\left(q\right)}$ respectively with a suitable tensor-field velocity and an effective quantum Hamiltonian density, and finally, the quantum phase-function $S^{\left(q\right)}$ with a four-scalar function of the form:

(25) $$S^{\left(q\right)}\left(g,\hat{g},r,s\right)\equiv S^{\left(q\right)}\left(g,\hat{g},r,s;P\right).$$

Thus, it follows that while the first one (i.e., Equation (23)) appears now modified (with respect to [2,3,44,45]) and carries the contribution of the graviton capture term $Q_{L}^{\left(q\right)}$, the second one represented by Equation (24) remains unchanged. In fact, $V_{\mu \nu }\equiv V_{\mu \nu }\left(g,s\right)$ is identified again with the quantum four-tensor velocity field:

(26) $$V_{\mu \nu }=\frac{1}{\alpha L}\frac{\partial S^{\left(q\right)}}{\partial g^{\mu \nu }},$$

and similarly, the effective quantum Hamiltonian density takes the formal structure given by the representation:

(27) $$H^{\left(q\right)}=\frac{1}{2\alpha L}\frac{\partial S^{\left(q\right)}}{\partial g^{\mu \nu }}\frac{\partial S^{\left(q\right)}}{\partial g_{\mu \nu }}+V_{QM}+V,$$

with V≡V(g,s) being the effective potential defined according to Equation (A3) and $V_{QM}\equiv V_{QM}\left(g,s\right)$ the Bohm effective quantum potential [52,53,54,55,56] of CQG-theory. The latter, in particular, recovers the customary expression:

(28) $$V_{QM}\equiv \frac{\hbar^{2}}{8\alpha L}\frac{\partial \ln\rho }{\partial g^{\mu \nu }}\frac{\partial \ln\rho }{\partial g_{\mu \nu }}-\frac{\hbar^{2}}{4\alpha L}\frac{\partial^{2}\rho }{\rho \partial g_{\mu \nu }\partial g^{\mu \nu }},$$

with $\rho =\rho (g,\hat{g},r,s)$ being now determined by the modified quantum continuity Equation (23). Thus, the prescription of $V_{QM}$ actually depends as in [3,45] on the precise determination of particular solutions of the same equation to be based once again on the so-called generalized Lagrangian path (GLP) representation of CQG-theory and of its CQG-wave equation.

## 3. GLP Representation of Non-Unitary CQG-Theory

To realize the goal indicated above, for its non-unitary generalization, CQG-theory can be conveniently cast in terms of the GLP-approach previously formulated in [3,45], which provides a stochastic trajectory-based representation of the CQG-wave equation. In the present section, this is used to determine an explicit representation for the quantum fluid fields $\left\{\rho ,S^{\left(q\right)}\right\}$ and in particular to show that the quantum PDF ρ admits now generally non-stationary Gaussian-like particular solutions. Here, for greater clarity, the key steps of the derivation are explicitly reported.

Originally developed in the context of non-relativistic quantum mechanics (QM) [15], the GLP-approach realizes a trajectory-based Lagrangian representation, which leaves unchanged the axiomatic basis of quantum theory. Thus, in particular, in the context of CQG-theory, this means that the manifestly-covariant character of the theory is naturally preserved. As in QM, the representation of CQG-theory is achieved by means of the introduction of suitable stochastic Lagrangian trajectories that span the corresponding configuration-space and are used to parametrize the quantum wave-function and its relevant quantum hydrodynamic equations. These are realized by the generalized Lagrangian paths (GLP), i.e., integral curves $\left\{\delta G_{L}\left(s\right)\equiv \delta G_{L\mu \nu }\left(r\left(s\right),s\right),\forall s\in I\right\}$, which are determined by the GLP-initial-value problem:

(29) $$\left\{\begin{array}{c} \frac{d}{ds}\delta G_{L\mu \nu }\left(s\right)=V_{\mu \nu }\left(G_{L}\left(s\right),\Delta g,r\left(s\right),s\right),\;\\ \delta G_{L\mu \nu }\left(s_{1}\right)=\delta g_{L\mu \nu }\left(s_{1}\right)-\Delta g_{\mu \nu }\left(s_{1}\right),\;\\ \delta g_{L\mu \nu }\left(s_{1}\right)\equiv \delta g_{L\mu \nu }^{\left(o\right)}. \end{array}\right.$$

Here, $\delta g_{L\mu \nu }\left(s\right)$ and $\delta G_{L\mu \nu }\left(s\right)$ identify the deterministic Lagrangian path (LP) and stochastic generalized Lagrangian path (GLP), $G_{L}\left(s\right)$ and $\delta G_{L\mu \nu }\left(s\right)$ are related by the equation:

(30) $$G_{L\mu \nu }\left(s\right)=\delta G_{L\mu \nu }\left(s\right)+{\hat{g}}_{\mu \nu }\left(r,s\right),$$

$\frac{d}{ds}$ denotes the covariant *s*-derivative (A5), while r(s) is an arbitrary geodesic trajectory with $s_{1}\geq s_{o}$ being in principle an arbitrary initial proper-time along it, which can always be required to be such that $s_{1}=s_{o}=0$. Furthermore, consistent with Equation (26), $\delta g_{\mu \nu }^{\left(o\right)}$ and $V_{\mu \nu }\left(G_{L}\left(s\right),\Delta g,r\left(s\right),s\right)$ denote respectively a deterministic initial tensor field and the quantum tensor velocity field:

(31) $$V^{\mu \nu }\left(G_{L}\left(s\right),\Delta g,r\left(s\right),s\right)=\frac{1}{\alpha L}\frac{\partial S^{\left(q\right)}\left(G_{L}\left(s\right),\Delta g,r\left(s\right),s;P\right)}{\partial \delta g_{L\mu \nu }},$$

where $\Delta g\equiv \left\{\Delta g_{\mu \nu }\right\}$ identifies the stochastic displacement four-tensor:

(32) $$\Delta g_{\mu \nu }\left(s\right)\equiv \delta g_{L\mu \nu }\left(s\right)-\delta G_{L\mu \nu }\left(s\right).$$

A formal solution of the GLP equations can be obtained in analytical form. To this aim, we preliminarily introduce the following compact notation:

(33) $$\left\{\begin{array}{c} \delta g_{L\nu }^{\alpha }\left(s\right)={\hat{g}}^{\mu \alpha }\left(r\left(s\right),s\right)\delta g_{L\mu \nu }\left(r\left(s\right),s\right),\;\\ \delta G_{L\nu }^{\alpha }\left(s\right)={\hat{g}}^{\mu \alpha }\left(r\left(s\right),s\right)\delta G_{L\mu \nu }\left(r\left(s\right),s\right),\;\\ \Delta g_{\nu }^{\alpha }\left(s\right)={\hat{g}}^{\mu \alpha }\left(r\left(s\right),s\right)\Delta g_{\mu \nu }\left(s\right),\;\\ V_{\nu }^{\alpha }\left(G_{L}\left(s\right),\Delta g,s\right)={\hat{g}}^{\mu \alpha }\left(r,s\right)V_{\mu \nu }\left(G_{L}\left(s\right),\Delta g,s\right). \end{array}\right.$$

Then, upon integration, Equation (29) actually delivers formal exact solutions both for $\delta g_{L\nu }^{\alpha }\left(s\right)$ and $\delta G_{L\nu }^{\alpha }\left(s\right)$, which are realized respectively by the initial-value problems:

(34) $$\left\{\begin{array}{c} \delta g_{L\nu }^{\alpha }\left(s\right)=\delta g_{L\nu }^{\alpha }\left(s_{o}\right)+\int_{s_{1}}^{s}ds^{\prime }V_{\nu }^{\alpha }\left(G_{L}\left(s^{\prime }\right),\Delta g,r\left(s^{\prime }\right),s^{\prime }\right),\;\\ \delta g_{L\nu }^{\alpha }\left(s_{o}\right)\equiv \delta g_{L\nu }^{\alpha (o)}, \end{array}\right.$$

and:

(35) $$\left\{\begin{array}{c} \delta G_{L\nu }^{\alpha }\left(s\right)=\delta G_{L\nu }^{\alpha }\left(s_{o}\right)+\int_{s_{1}}^{s}ds^{\prime }V_{\nu }^{\alpha }\left(G_{L}\left(s^{\prime }\right),\Delta g,r\left(s^{\prime }\right),s^{\prime }\right),\;\\ \delta G_{L\nu }^{\alpha }\left(s_{o}\right)=\delta g_{L\nu }^{\alpha }\left(s_{o}\right)-\Delta g_{\nu }^{\alpha }\left(s_{o}\right),\;\\ \delta g_{L\nu }^{\alpha }\left(s_{o}\right)\equiv \delta g_{L\nu }^{\alpha (o)}, \end{array}\right.$$

where:

(36) $$V_{\nu }^{\alpha }\left(G_{L}\left(s\right),\Delta g,r\left(s\right),s\right)=\frac{1}{\alpha L}\frac{\partial S^{\left(q\right)}\left(G_{L}\left(s\right),\Delta g,r\left(s\right),s;P\right)}{\partial \delta g_{L\alpha }^{\nu }\left(s\right)}.$$

These equations imply that the stochastic displacement four-tensor $\Delta g_{\nu }^{\mu }\left(s\right)$ is a constant, i.e., such that for all $s,s_{o}\in I$, $\Delta g_{\nu }^{\mu }\left(s\right)=\Delta g_{\nu }^{\mu }\left(s_{o}\right)\equiv \Delta g_{\nu }^{\mu }$, while $\delta g_{L\nu }^{\alpha }\left(s\right)$ and $\delta G_{L\nu }^{\alpha }\left(s\right)$ are related by means of the transformation:

(37) $$\delta G_{L\nu }^{\alpha }\left(s\right)=\delta g_{L\nu }^{\alpha }\left(s\right)-\Delta g_{\nu }^{\alpha }.$$

Equations (34) and (35) can also be represented in terms of the covariant components $\delta G_{L\mu \nu }\left(s\right)$ (and similarly, $\delta g_{L\mu \nu }\left(s\right)$), yielding:

(38) $$\begin{array}{c} \left.\delta G_{L\mu \nu }\left(s\right)={\hat{g}}_{\mu \alpha }\left(r\left(s\right),s\right)\delta G_{L\nu }^{\alpha }\left(s_{o}\right)+\right.\\ +{\hat{g}}_{\mu \alpha }\left(r\left(s\right),s\right)\int_{s_{1}}^{s}ds^{\prime }V_{\nu }^{\alpha }\left(G_{L}\left(s^{\prime }\right),\Delta g,r\left(s^{\prime }\right),s^{\prime }\right). \end{array}$$

Finally, upon invoking the condition ${\hat{g}}_{\mu \nu }={\hat{g}}_{\mu \nu }\left(r\right)$ corresponding to a stationary background metric tensor, the same equations reduce to the solution determined in [45], namely:

(39) $$\delta g_{L\mu \nu }\left(s\right)=\delta g_{L\mu \nu }\left(s_{o}\right)+\int_{s_{1}}^{s}ds^{\prime }V_{\mu \nu }\left(G_{L}\left(s^{\prime }\right),\Delta g,r\left(s^{\prime }\right),s^{\prime }\right).$$

One notices that the GLP initial-value problem (29) can be equivalently replaced with:

(40) $$\left\{\begin{array}{c} \frac{d}{ds}\delta G_{L\mu \nu }\left(s\right)=V_{\mu \nu }\left(G_{L}\left(s\right),\Delta g,s\right),\;\\ \delta G_{L\mu \nu }\left(s\right)=\delta g_{L\mu \nu }\left(s\right)-\Delta g_{\mu \nu },\;\\ \delta g_{L\mu \nu }\left(s\right)=\delta g_{L\mu \nu }, \end{array}\right.$$

with $\delta g_{L\mu \nu }$ prescribing now the initial condition (associated with the deterministic Lagrangian path). Equation (40) admits the formal solution:

(41) $$\left\{\begin{array}{c} \delta G_{L\nu }^{\alpha }\left(s\right)=\delta G_{L\nu }^{\alpha }\left(s_{1}\right)+\int_{s_{1}}^{s}ds^{\prime }V_{\nu }^{\alpha }\left(G_{L}\left(s^{\prime }\right),\Delta g,r\left(s^{\prime }\right),s^{\prime }\right),\;\\ \delta G_{L\nu }^{\alpha }\left(s\right)=\delta g_{L\nu }^{\alpha }-\Delta g_{\nu }^{\alpha }, \end{array}\right.$$

while correspondingly:

(42) $$\left\{\begin{array}{c} \delta g_{L\nu }^{\alpha }\left(s\right)=\delta g_{L\nu }^{\alpha }\left(s_{1}\right)+\int_{s_{1}}^{s}ds^{\prime }V_{\nu }^{\alpha }\left(G_{L}\left(s^{\prime }\right),\Delta g,r\left(s^{\prime }\right),s^{\prime }\right),\;\\ \delta g_{L\nu }^{\alpha }\left(s\right)=\delta g_{L\nu }^{\alpha }. \end{array}\right.$$

As a consequence, the stochastic displacement four-tensor defined by Equation (32) can also be equivalently represented as:

(43) $$\Delta g_{\nu }^{\mu }\left(s\right)\equiv \delta g_{L\nu }^{\mu }-\delta G_{L\nu }^{\mu }\left(s\right),$$

where $\delta g_{L\nu }^{\mu }\equiv g_{L\nu }^{\mu }-{\hat{g}}_{\nu }^{\mu }\left(r,s\right)$ is considered prescribed and $\delta G_{L\nu }^{\mu }\left(s\right)$ is a function of the proper-time *s*. Then, introducing as in [45] the Lagrangian derivative realized by the operator:

(44) $$\frac{D}{Ds}\equiv {\left.\frac{d}{ds}\right|}_{\delta g_{L\mu \nu }\left(s\right)}+V_{\nu }^{\mu }\left(G_{L}\left(s\right),\Delta g,r,s\right)\frac{\partial }{\partial \delta g_{L\nu }^{\mu }},$$

and upon denoting ${\left.\frac{d}{ds}\right|}_{\delta g_{L\nu }^{\mu }\left(s\right)}\equiv \frac{d}{ds}$ and invoking also Equation (40), one finds that the differential identity:

(45) $$\frac{D}{Ds}\Delta g_{\nu }^{\mu }\left(s\right)=V_{\nu }^{\mu }\left(G_{L}\left(s\right),\Delta g,r,s\right)-V_{\nu }^{\mu }\left(G_{L}\left(s\right),\Delta g,r,s\right)\equiv 0$$

necessarily holds.

The GLP-approach permits the construction of analytical polynomial solutions for the quantum phase-function $S^{\left(q\right)}\left(G_{L}\left(s\right),\Delta g,r,s\right),$ to be represented in terms of a “harmonic” decomposition. This feature remains unchanged with respect to the original formulation of CQG-theory achieved in [3,45] and is obtained by introducing the second-degree polynomial representation of the form:

(46) $$S^{\left(q\right)}\left(G_{L}\left(s\right),\Delta g,r,s;P\right)=\frac{a_{pq}^{\alpha \beta }\left(s\right)}{2}\Delta g_{\alpha \beta }\Delta g^{pq}+b_{\alpha \beta }\left(s\right)\Delta g^{\alpha \beta }+c\left(s\right).$$

Here, $a_{\mu \nu }^{\alpha \beta }\left(s\right)$, $b_{\mu \nu }\left(s\right)$, and c(s) denote respectively suitable real four-tensors and a four-scalar function of *s* to be determined in terms of the quantum H-J Equation (24) recalled above. In particular, consistent with [45] and upon denoting $\delta_{pq}^{\alpha \beta }\equiv \delta_{p}^{\alpha }\delta_{q}^{\beta }$, the tensor coefficients $a_{pq}^{\alpha \beta }\left(s\right)$ are taken in the form:

(47) $$a_{pq}^{\alpha \beta }\left(s\right)=\frac{1}{2}\left[a_{\left(o\right)}\left(s\right)\delta_{pq}^{\alpha \beta }+a_{\left(1\right)}\left(s\right){\hat{g}}_{pq}\left(s\right){\hat{g}}^{\alpha \beta }\left(s\right)\right],$$

with $a_{\left(o\right)}\left(s\right)$ and $a_{\left(1\right)}\left(s\right)$ being appropriate four-scalar functions. Since:

(48) $$\left\{\begin{array}{c} \delta_{pq}^{\alpha \beta }\Delta g_{\alpha \beta }\Delta g^{pq}=\Delta g_{\alpha \beta }\Delta g^{\alpha \beta },\;\\ {\hat{g}}_{pq}\left(s\right){\hat{g}}^{\alpha \beta }\left(s\right)\Delta g_{\alpha \beta }\Delta g^{pq}=\Delta g_{\alpha }^{\alpha }\Delta g_{\beta }^{\beta }, \end{array}\right.$$

from Equation (46), it follows that:

(49) $$\alpha_{pq}^{\alpha \beta }\left(s\right)\Delta g_{\alpha \beta }\Delta g^{pq}=\frac{1}{2}\left[a_{\left(o\right)}\left(s\right)\Delta g_{\alpha \beta }\Delta g^{\alpha \beta }+a_{\left(1\right)}\left(s\right)\Delta g_{\alpha }^{\alpha }\Delta g_{\beta }^{\beta }\right],$$

and therefore:

(50) $$S^{\left(q\right)}\left(G_{L}\left(s\right),\Delta g,s\right)=\frac{1}{4}\left[a_{\left(o\right)}\left(s\right)\Delta g_{\alpha \beta }\Delta g^{\alpha \beta }+a_{\left(1\right)}\left(s\right)\Delta g_{\alpha }^{\alpha }\Delta g_{\beta }^{\beta }\right]+b_{\alpha \beta }\left(s\right)\Delta g^{\alpha \beta }+c\left(s\right).$$

On the same grounds, the effective quantum Hamiltonian density (27) can equivalently be represented as:

(51) $$H^{\left(q\right)}=\frac{1}{2\alpha L}\frac{\partial S^{\left(q\right)}}{\partial \delta g_{L\nu }^{\mu }}\frac{\partial S^{\left(q\right)}}{\partial \delta g_{L\mu }^{\nu }}+V_{QM}+V,$$

where:

(52) $$\frac{\partial S^{\left(q\right)}}{\partial \delta g_{\nu }^{\mu }}=p\left(s\right)\left[a_{\left(o\right)}\left(s\right)\Delta g_{\mu }^{\nu }+a_{\left(1\right)}\left(s\right)\delta_{\mu }^{\nu }\Delta g_{\beta }^{\beta }\right]+p\left(s\right)b_{\mu }^{\nu }\left(s\right).$$

The quantum-modified Einstein field equations proposed in [3] are then recovered by requiring the identical validity of the extremal equation:

(53) $${\left.\frac{\partial }{\partial \Delta g^{\mu \nu }}\left[V_{o}\left(g+\Delta g\right)+V_{QM}\left(g,s\right)\right]\right|}_{\Delta g=0}=0.$$

Thanks to the harmonic decomposition introduced above (see Equation (46)), GLP-Gaussian particular solutions of the quantum PDF can be determined to achieve particular solutions of the non-unitary generalization of the quantum continuity equation, which is realized by Equation (23). In fact, in terms of the GLP-representation, the same equation can now be explicitly solved to get for the effective PDF $\rho =\rho (G_{L}\left(s\right),\hat{g}\left(s\right),\Delta g,r\left(s\right),s)$ the non-stationary Gaussian-like solution of the form:

(54) $$\begin{array}{c} \left.\rho \left(G_{L}\left(s\right),\hat{g}\left(s\right),\Delta g,r\left(s\right),s\right)=\rho \left(G_{L}\left(s_{o}\right),\hat{g}\left(s_{o}\right),\Delta g\left(s_{o}\right),r\left(s_{o}\right),s_{o}\right)\right.\\ \eta \left(s\right)\exp\left\{-\int_{s_{o}}^{s}ds^{\prime }\frac{\partial V_{\nu }^{\mu }\left(G_{L}\left(s^{\prime }\right),\Delta g,r\left(s^{\prime }\right),s^{\prime }\right)}{\partial g_{L\nu }^{\mu }\left(s^{\prime }\right)}\right\}, \end{array}$$

where the proper-time dependent exponential factor:

(55) $$\eta \left(s\right)\equiv \exp\left\{\int_{s_{o}}^{s}ds^{\prime }f\left(r\left(s^{\prime }\right),s^{\prime }\right)\Theta \left(\varepsilon -\Delta \right)\right\}$$

carries the new non-stationary modification produced by the graviton capture term $f\left(r\left(s^{\prime }\right),s^{\prime }\right)\Theta \left(\varepsilon -\Delta \right)$ appearing in the quantum continuity equation. Notice that in principle, the magnitude of the integral $\int_{s_{o}}^{s}ds^{\prime }f\left(r\left(s^{\prime }\right),s^{\prime }\right)\Theta \left(\varepsilon -\Delta \right)$ is necessarily bounded for s→∞ (and hence, η(s) as well). However, it is obvious that in order for the effective quantum PDF to remain globally non-vanishing and finite, the double inequality:

(56) $$0<\left|\lim_{s\to \infty }\int_{s_{o}}^{s}ds^{\prime }f\left(r\left(s^{\prime }\right),s^{\prime }\right)\Theta \left(\varepsilon^{2}-\Delta s^{2}\right)\right|<\infty$$

should hold as well. Therefore, if the source function f(r(s),s) remains strictly positive or negative, this means that in the limit s→+∞, the same function must necessarily tend to zero, so that:

(57) $$\lim_{s\to +\infty }\left|f(r(s),s)\right|=0.$$

The rest of the notations remain unchanged with respect to the solution earlier determined in [3,45]. Thus, in particular, the coefficient $\rho (G_{L}\left(s_{o}\right),\hat{g}\left(s_{o}\right),\Delta g\left(s_{o}\right),r\left(s_{o}\right),s_{o})$ identifies the initial PDF. In terms of the signature parameter θ≡±, the function:

(58) $$\begin{array}{ccc} \rho (G_{L}\left(s_{o}\right),\Delta g\left(s_{o}\right),r\left(s_{o}\right),s_{o}) & \equiv & \frac{1}{\pi^{5}r_{th}^{10}}\exp\left\{-\frac{{\left(\Delta g\left(s_{o}\right)+\theta \hat{g}\left(s_{o}\right)\right)}^{2}}{r_{th}^{2}}\right\}\\ & \equiv & \rho_{G}\left(\Delta g\left(s_{o}\right)+\theta \hat{g}\left(s_{o}\right)\right) \end{array}$$

identifies an initial shifted Gaussian PDF, with $\hat{g}\left(s\right)\equiv \hat{g}\left(r\left(s\right),s\right)$ and $\hat{g}\left(s_{o}\right)\equiv \hat{g}\left(r\left(s_{o}\right),s_{o}\right)$ denoting a generally non-stationary background metric tensor and its initial value at the initial proper-time $s_{o}$ evaluated along an observer’s geodesic curve. In particular, with the validity of the polynomial decomposition (50) for the quantum phase function $S^{\left(q\right)}\left(G_{L}\left(s\right),\Delta g,r,s\right)$, the four-scalar function $\frac{\partial V_{\mu \nu }\left(G_{L}\left(s^{\prime }\right),\Delta g,s^{\prime }\right)}{\partial g_{L\mu \nu }\left(s^{\prime }\right)}$ is found to be a function of proper-time only. More precisely, it takes the form:

(59) $$\frac{\partial V_{\nu }^{\mu }\left(G_{L}\left(s^{\prime }\right),\Delta g,r\left(s^{\prime }\right)s^{\prime }\right)}{\partial g_{L\nu }^{\mu }\left(s^{\prime }\right)}\equiv 16p^{2}\left(s^{\prime }\right)a\left(s^{\prime }\right),$$

where for $s^{\prime }=s$, p(s) is given by:

(60) $$p\left(s\right)=\frac{1}{{\left(1+\frac{2}{\alpha L}\int_{s_{o}}^{s}ds^{\prime }a\left(s^{\prime }\right)\right)}^{1/2}},$$

and the four-scalar function $a(s^{\prime })$ is prescribed by requiring:

(61) $$a\left(s^{\prime }\right)=\frac{1}{2}\left[a_{\left(o\right)}\left(s^{\prime }\right)+a_{\left(1\right)}\left(s^{\prime }\right)\right]$$

(or equivalently, $a\left(s\right)\equiv \frac{1}{4}a_{q\beta }^{p\alpha }\left(s\right)\delta_{p}^{q}\;\delta_{\alpha }^{\beta }$). In addition, one notices that here, both $r_{th}^{2}$ and ${\left(\Delta g+\theta \hat{g}\left(s_{o}\right)\right)}^{2}$ are four-scalars and:

(62) $${\left(\Delta g\left(s_{o}\right)+\theta \hat{g}\left(s_{o}\right)\right)}^{2}\equiv {\left(\Delta g\left(s_{o}\right)+\theta \hat{g}\left(s_{o}\right)\right)}_{\mu \nu }{\left(\Delta g\left(s_{o}\right)+\theta \hat{g}\left(s_{o}\right)\right)}^{\mu \nu },$$

and $r_{th}^{2}$ is a constant independent of both the four-position $r^{\mu }$ and the proper-time *s*. In particular, the validity of the invariance property:

(63) $${\left(\Delta g\left(s_{o}\right)+\theta \hat{g}\left(s_{o}\right)\right)}^{2}={\left(\Delta g\left(s\right)+\theta \hat{g}\left(s\right)\right)}^{2}$$

remains preserved for arbitrary $s,s_{o}\in I$, so that as a consequence:

(64) $$\rho_{G}\left(\Delta g\left(s_{o}\right)+\theta \hat{g}\left(s_{o}\right)\right)=\rho_{G}\left(\Delta g+\theta \hat{g}\left(s\right)\right).$$

It follows that Equation (54) takes the form:

(65) $$\rho \left(G_{L}\left(s\right),\Delta g,s\right)=\rho_{G}\left(\Delta g+\theta \hat{g}\left(s\right)\right)\eta \left(s\right)\exp\left\{-16\int_{s_{o}}^{s}ds^{\prime }p^{2}\left(s^{\prime }\right)a\left(s^{\prime }\right)\right\},$$

which indeed recovers also in the present case a Gaussian particular solution for the quantum PDF. The key difference with respect to the conventional formulation of CQG-theory (earlier reported in [3,45]) is therefore the appearance of the non-stationary and non-unitary correction factor η(s) prescribed according to Equation (55). In particular, in the case that the source term corresponds to a quantum sink that realizes an effective local capture mechanism for gravitons sufficiently close to the EH, this requires the inequality $f\left(r\left(s^{\prime }\right)\right)\Theta \left(\varepsilon -\Delta \right)<0$ to hold. This implies for the effective PDF the non-unitary normalization condition indicated above (see Equation (22)).

Although in principle, both Gaussian solutions corresponding to θ=+ and θ=− are admissible from the mathematical point of view, only the one obtained from Equation (54) by setting θ≡− is physically acceptable in the present context, so that the initial shifted Gaussian PDF is taken in the following of the form:

(66) $$\begin{array}{c} \left.\rho \left(G_{L}\left(s_{o}\right),\Delta g\left(s_{o}\right),r\left(s_{o}\right),s_{o}\right)\equiv \rho_{G}\left(\Delta g\left(s_{o}\right)-\hat{g}\left(s_{o}\right)\right)\right.\\ =\frac{1}{\pi^{5}r_{th}^{10}}\exp\left\{-\frac{{\left(\Delta g\left(s_{o}\right)-\hat{g}\left(s_{o}\right)\right)}^{2}}{r_{th}^{2}}\right\}\equiv \rho_{G}\left(\Delta g\left(s_{o}\right)-\hat{g}\left(s_{o}\right)\right), \end{array}$$

where (as shown in [3,45]) one finds that by construction, the initial quantum PDF $\rho_{G}\left(\Delta g\left(s_{o}\right)-\hat{g}\left(s_{o}\right)\right)$ satisfies the integral identity:

(67) $$\rho_{G}\left(\Delta g\left(s_{o}\right)-\hat{g}\left(s_{o}\right)\right)=\rho_{G}\left(\Delta g\left(s\right)-\hat{g}\left(s\right)\right),$$

where the rhs denotes:

(68) $$\rho_{G}\left(\Delta g\left(s\right)-\hat{g}\left(s\right)\right)\equiv \frac{1}{\pi^{5}r_{th}^{10}}\exp\left\{-\frac{{\left(\Delta g\left(s\right)-\hat{g}\left(s\right)\right)}^{2}}{r_{th}^{2}}\right\}.$$

The choice of the initial PDF indicated above (see Equation (66)) has a physical basis. In fact, according to the emergent gravity picture inherent in the GLP formulation of CQG-wave equation, it warrants that the GLP-quantum/stochastic expectation value of the stochastic displacement four-tensor $\Delta g_{\mu \nu }$ recovers the correct signature of the background metric tensor. For definiteness, let us adopt here the notations of the GLP-approach [45]. In such a context, one notices that thanks to the differential identity:

(69) $$d\left(\delta G\left(s\right)\right)\rho \left(G_{L}\left(s\right),\Delta g,s\right)=d\left(\Delta g\right)\eta \left(s\right)\rho \left(G_{L}\left(s_{o}\right),\Delta g\left(s_{o}\right),r\left(s_{o}\right),s_{o}\right)$$

the normalization condition for the quantum PDF $\rho (G_{L}\left(s\right),\Delta g,s)$ yields also thanks to (66):

(70) $$\begin{array}{ccc} \langle 1\rangle & = & \int_{U_{g}}d\left(\delta G\right)\rho \left(G_{L}\left(s\right),\Delta g,s\right)\\ & = & \eta \left(s\right)\int_{U_{g}}d\left(\Delta g\left(s_{o}\right)\right)\rho_{G}\left(\Delta g\left(s_{o}\right)-\hat{g}\left(s_{o}\right)\right)=\eta \left(s\right), \end{array}$$

with $\langle A\rangle$ for A=1 identifying the quantum expectation value associated with a dynamical variable *A*. Notice that to perform the integration in Equation (70), once letting $\Delta g_{\mu \nu }\left(s_{o}\right)-{\hat{g}}_{\mu \nu }\left(s_{o}\right)\equiv$ $\Delta_{1}g_{\mu \nu }\left(s_{o}\right),$ one obtains equivalently:

(71) $$\langle 1\rangle =\eta \left(s\right)\int_{U_{g}}d\left(\Delta_{1}g\left(s_{o}\right)\right)\rho_{G}\left(\Delta_{1}g\left(s_{o}\right)\right).$$

Hence, for consistency, one has to require that by construction, the inequality:

(72) $$\Delta_{1}g_{\mu \nu }\left(s_{o}\right)\Delta_{1}g^{\mu \nu }\left(s_{o}\right)\equiv \Delta_{1}g_{\mu }^{\nu }\left(s_{o}\right)\Delta_{1}g_{\nu }^{\mu }\left(s_{o}\right)>0$$

holds identically in $U_{g}$. Such a condition is always satisfied provided $\Delta_{1}g_{\mu \nu }\left(s_{o}\right)$ is symmetric. As a consequence, thanks to the integral identity (67) and noting that $d\left(\Delta g(s_{o})\right)=d\left(\Delta g\left(s\right)\right)$, it follows that the expectation value of the stochastic tensor field $\Delta g_{\mu \nu }$ is just:

(73) $$\langle \Delta g_{\mu \nu }\rangle =\langle 1\rangle \int_{U_{g}}d\left(\Delta g\left(s\right)\right)\rho_{G}\left(\Delta g\left(s\right)-\hat{g}\left(s\right)\right)\Delta g_{\mu \nu }=\langle 1\rangle {\hat{g}}_{\mu \nu }\left(r,s\right),$$

which implies:

(74) $$\frac{\langle \Delta g_{\mu \nu }\rangle }{\langle 1\rangle }={\hat{g}}_{\mu \nu }\left(r,s\right),$$

the lhs identifying the corresponding normalized quantum expectation value (of $\Delta g_{\mu \nu }$). Hence, as in [45], the background tensor field ${\hat{g}}_{\mu \nu }\left(r,s\right)$ is uniquely determined by a suitable expectation value of $\Delta g_{\mu \nu }$ identified with its normalized quantum expectation value $\langle \Delta g_{\mu \nu }\rangle /\langle 1\rangle$. Therefore, the non-unitary realization of CQG-theory preserves the emergent-gravity interpretation of CQG-theory, i.e., the so-called second-type paradigm of emergent gravity [3], according to which, the background metric tensor should emerge as a suitable statistical average of the corresponding quantum field. For an exhaustive discussion of the subject occurring in the unitary case for which identically $\langle 1\rangle =1$, we refer also to [45] (see in particular Proposition 3 and the related comments reported in the same reference).

Finally, it is worth mentioning that it is possible to prove in an analogous way (details are omitted for brevity) that the connection between the CQG-wave equation and the quantum-modified Einstein field equations earlier pointed out in [3] remains still preserved also in the present case.

## 4. CQG Boltzmann–Shannon Entropy

In this section, the entropic properties of the CQG-theory in the presence of a localized quantum sink are addressed by first defining the notion of quantum entropy for the quantum gravitational field and of the corresponding entropy production rate, which pertain to the theory of covariant quantum gravity. The goal is two-fold. First, in this section, we intend to show that in the context of CQG-theory, quantum entropy can be identified with the quantum expectation value of a suitable continuous four-scalar quantum phase-function, which is identified with the quantum entropy density:

(75) $$E_{BS}\left(\rho \right)\equiv -\ln\rho ,$$

with ρ=ρ(g,s) being the relevant quantum PDF. Such a prescription is shown to coincide formally with the customary prescription of quantum differential entropy adopted in quantum theory, and in particular in quantum mechanics (see for example [45]). Hence, as such, it coincides necessarily with a real four-scalar field, which is locally defined everywhere in the background space-time $\left\{Q^{4},\hat{g}\right\}$ and may depend also explicitly on proper time *s*. In the present context, such a formulation is made possible by the explicit determination of the quantum PDF. Second, in the subsequent section, the issue of the possible validity of local, either strong or weak, H-theorems for the same entropy will be investigated.

To start with, we recall the notion of quantum differential entropy. The approach is well known in the context of non-relativistic QM, where the notion of quantum statistical entropy was first developed (for a related discussion, see for example [45]). However, the same approach can in principle be formulated for an arbitrary quantum theory that is endowed with a corresponding quantum PDF ρ defined on a suitable quantum configuration space (*D*), provided the same PDF satisfies an appropriate quantum hydrodynamic equation identified with the corresponding quantum continuity equation. Such a notion of quantum entropy coincides therefore with the quantum expectation value:

(76) $$S_{BS}\left(\rho \right)\equiv \langle E_{BS}\left(\rho \right)\rangle ,$$

with $E_{BS}\left(\rho \right)$ being the quantum phase function (75). The same expectation value $\langle E_{BS}\left(\rho \right)\rangle$ is defined here as the integral performed on the configuration space *D* and weighted with respect to the corresponding quantum PDF ρ. Hence, it is actually realized by the corresponding *quantum Boltzmann–Shannon* (BS) *entropy* for the same PDF, namely:

(77) $$\langle E_{BS}\left(\rho \right)\rangle =-\int_{D}d\Omega \rho E_{BS}\left(\rho \right),$$

dΩ being the related configuration-space volume element. In other words, $S_{BS}\left(\rho \right)$ is related to the ignorance of the same quantum PDF ρ, i.e., in turn, a measure of the ignorance of the knowledge of the corresponding quantum state ψ.

In the context of CQG theory, the configuration space *D* and the related PDF ρ are identified respectively with the *g*-space $U_{g}$ and the corresponding *g*-space effective quantum probability density. Specifically, in the framework of GLP representation, the quantum PDF ρ is represented as $\rho (G_{L}\left(s\right),\Delta g,s)$ defined above by Equation (65), while the entropy functional will take the form:

(78) $$S_{BS}\left(\rho (G_{L}\left(s\right),\Delta g,s)\right)=\langle E_{BS}\left(G_{L}\left(s\right),\Delta g,s\right)\rangle =-\int_{U_{g}}d\left(\delta G\right)\rho \left(G_{L}\left(s\right),\Delta g,s\right)E_{BS}\left(G_{L}\left(s\right),\Delta g,s\right),$$

to be denoted as *CQG Boltzmann–Shannon entropy*. In terms of the differential identity (69), this gives therefore:

(79) $$S_{BS}\left(\rho (G_{L}\left(s\right),\Delta g,s)\right)=-\eta \left(s\right)\int_{U_{g}}d\left(\Delta g\left(s_{o}\right)\right)\rho \left(G_{L}\left(s_{o}\right),\Delta g,s_{o}\right)\ln\rho \left(G_{L}\left(s\right),\Delta g,s\right).$$

Hence, introducing the *normalized CQG BS entropy*:

(80) $${\bar{S}}_{BS}\left(\rho (G_{L}\left(s\right),\Delta g,s)\right)\equiv \frac{S_{BS}\left(\rho (G_{L}\left(s\right),\Delta g,s)\right)}{\langle 1\rangle },$$

it follows that this is just:

(81) $$\begin{array}{c} \left.{\bar{S}}_{BS}\left(\rho (G_{L}\left(s\right),\Delta g,s)\right)\equiv \frac{S_{BS}\left(\rho (G_{L}\left(s\right),\Delta g,s)\right)}{\eta (s)}\right.\\ \left.=\int_{U_{g}}d\left(\Delta g\left(s_{o}\right)\right)\rho_{G}\left(\Delta g\left(s_{o}\right)-\hat{g}\left(s_{o}\right)\right)\ln\rho \left(G_{L}\left(s\right),\Delta g,s\right).\right. \end{array}$$

Differentiation under the integral with respect to the proper time *s* of the same functional yields therefore the corresponding *normalized CQG BS entropy production rate*. Upon invoking again the integral identity (67), one finds therefore:

(82) $$\frac{\partial {\bar{S}}_{BS}\left(\rho (s)\right)}{\partial s}=-\int_{U_{g}}d\left(\Delta g\left(s\right)\right)\rho_{G}\left(\Delta g\left(s\right)-\hat{g}\left(s\right)\right)\frac{\partial }{\partial s}\ln\rho \left(G_{L}\left(s\right),\Delta g,s\right),$$

where the partial derivative under the integral can be explicitly evaluated in terms of $\rho (G_{L}\left(s\right),\Delta g,s)$. Thanks to Equations (54) and (55), this delivers:

(83) $$\frac{\partial }{\partial s}\ln\rho \left(G_{L}\left(s\right),\Delta g,s\right)=f\left(r\left(s\right),s\right)\Theta \left(\varepsilon -\Delta \right)-\frac{\partial V_{\nu }^{\mu }\left(G_{L}\left(s^{\prime }\right),\Delta g,r\left(s^{\prime }\right),s^{\prime }\right)}{\partial g_{L\nu }^{\mu }\left(s^{\prime }\right)},$$

where in the case of vacuum, the contribution of the divergence term (the second term on the rhs) is determined by Equation (59). As a consequence, in the case of a vacuum, one obtains finally for the normalized entropy production rate the expression:

(84) $$\frac{\partial {\bar{S}}_{BS}\left(\rho (s)\right)}{\partial s}=-Q_{L}^{\left(q\right)}+Q_{V}^{\left(q\right)},$$

where $Q_{L}^{\left(q\right)}$ and $Q_{V}^{\left(q\right)}$ denote respectively the graviton capture term (18) and the quantum vacuum term:

(85) $$Q_{V}=16p^{2}\left(s\right)\frac{a(s)}{\alpha L},$$

with $p^{2}\left(s\right)$ being the real factor:

(86) $$p^{2}\left(s\right)=\frac{1}{1+\frac{2}{\alpha L}\int_{s_{o}}^{s}ds^{\prime }a\left(s^{\prime }\right)}.$$

Equation (84) is the main result of the paper. It represents the space-time normalized entropy production rate associated with the quantum (graviton) probability density arising in the context of CQG-theory. We notice that in the case of a vacuum, it is determined by two different contributions: (a) by a quantum capture term, which may arise only in the vicinities of the EH and is represented by the first term on the rhs of Equation (84). In particular, in the case of a quantum sink, the first term is positive; (b) a vacuum-field contribution carried by the divergence term of the tensor velocity field, namely $\frac{\partial V_{\nu }^{\mu }}{\partial g_{L\nu }^{\mu }\left(s^{\prime }\right)}$. Regarding its signature, one notices that although by construction, $1+\frac{2}{\alpha L}\int_{s_{o}}^{s}ds^{\prime }a\left(s^{\prime }\right)$ is necessarily strictly >0, the four-scalar function a(s) need not be so. This implies that the normalized BS entropy production rate does not have generally a defined sign, since both the scalar factors f(r(s),s) and a(s) can in principle carry either positive or negative values. This means that in general, it is not possible to establish an H-theorem holding for a finite proper time *s*. Nevertheless, particular cases can be distinguished, as discussed below.

## 5. Entropic Properties and the Search of H-Theorems for Quantum Gravity

In this section, the behavior of the BS entropy production rate associated with the quantum probability density of space-time occurring in vacuum and in the presence of a localized quantum sink is investigated. In detail, the following examples are considered:

- *Case #1*: Entropic behavior in the EH-capture domain.

Let us examine first the behavior in the vicinity of a black hole event horizon, i.e., in the EH-capture domain, where irreversible entropic effects, which are carried by the non-unitary behavior of the quantum PDF, are expected to appear. We intend to show that in the presence of a quantum sink, a strong H-theorem may hold, and the normalized entropy production rate is larger than zero at least for a finite proper-time interval. This warrants in turn the monotonic increase with respect to proper-time *s* of the corresponding normalized quantum entropy.

If the background solution is taken to be that of a classical black hole having an event horizon separating internal and external domains of the black hole, such a regime is expected to occur in a subset of the external domain sufficiently close to the event horizon where the condition $ds^{2}=0$ occurs (see the classification (7)). Notice that when the EH itself is approached, it occurs that ds→0, and hence, proper-time itself tends to a constant $s\to s_{o}$. In the same limit:

(87) $$\lim_{s\to s_{o}}a\left(s\right)=0,$$

while in the EH-capture domain:

(88) a(s)≅0.

This implies that in such a domain, the background space-time takes the form of a stationary background $\hat{g}\left(r\right)\equiv \left\{{\hat{g}}_{\mu \nu }\left(r\right)\right\}$. On the other hand, one notices that when $s\to s_{o}$, the quantum PDF should reduce identically to a constant, precisely:

(89) $$\rho \left(G_{L}\left(s\right),\Delta g,s\right)\to \rho_{G}\left(\Delta g-\hat{g}\left(s_{o}\right)\right),$$

while its covariant *s*-derivative remains non-vanishing:

(90) $$\frac{d\rho (G_{L}\left(s\right),\Delta g,s)}{ds}\to \rho_{G}\left(\Delta g-\hat{g}\left(s_{o}\right)\right).$$

In turn, Equation (87) implies that the quantum vacuum term $Q_{V}\left(s\right)$ prescribed by Equation (85) must vanish as well, namely:

(91) $$\lim_{s\to s_{o}}Q_{V}\left(s\right)=0.$$

As a consequence, one expects that in the EH-capture domain, only the graviton capture term (18) can contribute to the normalized BS entropy production rate (84). The latter reduces therefore to the expression:

(92) $$\frac{\partial {\bar{S}}_{BS}\left(\rho (s)\right)}{\partial s}≅-Q_{L}^{\left(q\right)},$$

with $Q_{L}^{\left(q\right)}$ taking into account the graviton capture term, i.e., the loss effect carried by the quantum PDF and assumed non-vanishing for arbitrary finite proper-times *s*. On the other hand, since by assumption, $Q_{L}^{\left(q\right)}<0$, it follows that the two inequalities:

(93) $$\left\{\begin{array}{c} \frac{\partial {\bar{S}}_{BS}\left(\rho (G_{L}\left(s\right),\Delta g,s)\right)}{\partial s}>0,\;\\ \lim_{\begin{array}{c} s\to s_{o}\\ \rho \to \rho_{G} \end{array}}\frac{\partial {\bar{S}}_{BS}\left(\rho (G_{L}\left(s\right),\Delta g,s)\right)}{\partial s}>0, \end{array}\right.$$

necessarily hold. The same inequalities imply the strict positivity of the normalized entropy production rate both in the EH-capture domain and on the EH itself. Hence, in both cases, a *strong H-theorem* is established, warranting the irreversible monotonically-increasing proper-time behavior of the normalized BS entropy ${\bar{S}}_{BS}\left(\rho (G_{L}\left(s\right),\Delta g,s)\right)$. This means, as a result, that a loss of information about the quantum gravitational state occurs, which arises because of the property of non-unitarity, i.e., non-conservation of quantum probability, introduced in the CQG-wave equation and the corresponding quantum continuity equation (see respectively Equations (19) and (74)).

- *Case #2*: Entropic behavior for stationary background in the external EH domain.

In this case, we intend to show that for stationary background space-time, the entropy production rate vanishes identically so that a constant H-theorem holds.

In the external EH domain, quantum unitarity is preserved by assumption so that the time-dependent exponential factor (defined by Equation (55)) satisfies identically the constraint η(s)=1. As a consequence, Equation (84) reduces to:

(94) $$\frac{\partial S_{BS}\left(\rho (s)\right)}{\partial s}=Q_{V}^{\left(q\right)}\left(s\right),$$

with $S_{BS}\left(\rho (s)\right)$ denoting the CQG-BS entropy (78). Nevertheless, despite unitarity, $\frac{\partial S_{BS}\left(\rho (s)\right)}{\partial s}$ is generally non-zero. In fact, the signature of $Q_{V}^{\left(q\right)}\left(s\right)$ remains generally not “a priori” defined. An exception, however, occurs in the case of a stationary background space-time $\hat{g}\left(r\right)\equiv \left\{{\hat{g}}_{\mu \nu }\left(r\right)\right\}$. In such a case, one can show (see [3,45]) that by construction, a(s)≡0 must hold. This implies, as a result, that:

(95) $$\frac{\partial S_{BS}\left(\rho (s)\right)}{\partial s}=0,$$

i.e., that a *constant H-theorem* holds identically for arbitrary stationary background space-times. In other words, in the external EH domain, the quantum BS entropy remains always constant when stationary solutions of the quantum-modified Einstein field equation introduced in [3] are considered. According to the same reference, such a property is fulfilled if the cosmological constant Λ is assumed independent of proper-time *s*.

- *Case #3*: Asymptotic entropic proper-time behavior.

Finally, let us consider the general case of a non-stationary background space-time, namely of the form (3). We intend to show that for s→+∞, the entropy (asymptotic) behavior requires it to become constant, so that the corresponding entropy production rate must vanish. This implies therefore the validity of an asymptotic constant H-theorem for the quantum BS entropy of space-time. More precisely, the goal is to show that in the limit s→∞, the asymptotic limits:

(96) $$\left\{\begin{array}{c} \lim_{s\to +\infty }\frac{\partial {\bar{S}}_{BS}\left(\rho (s)\right)}{\partial s}=0,\;\\ \lim_{s\to +\infty }\frac{\partial S_{BS}\left(\rho (s)\right)}{\partial s}=0, \end{array}\right.$$

hold respectively both in the EH-capture domain and in the external EH domain. The first limit is a consequence of the requirement (56) set above implying necessarily that:

(97) $$\lim_{s\to +\infty }f\left(r\left(s\right),s\right)\Theta \left(\varepsilon -\Delta \right)=0.$$

The second one, instead, follows from the asymptotic behavior of the four-scalar function investigated in [3], which can be shown to require:

(98) $$\lim_{s\to +\infty }a\left(s\right)=0.$$

The implication is therefore that both in the EH-capture domain and in the external EH domain, an asymptotic *constant H-theorem* holds.

The discussions reported above are relevant for the investigation of vacuum solutions of the quantum-modified Einstein field equations and the treatment of event horizons, such as the one characteristic of the de Sitter cosmological solution, which can be characterized by the presence of localized quantum sinks. The implication is the validity in all *Cases #1–#3* indicated above of either strong or constant H-theorems, a result which, incidentally, is consistent with the interpretation of quantum entropy as a measure of ignorance of the quantum state ψ.

## 6. Conclusions

The entropic properties of the gravitational field, and more generally the thermodynamic ones, investigated in previous literature have been traditionally associated with the mathematical properties of classical black hole solutions, i.e., in the framework of general relativity. In particular, beginning with the pioneering work of Bekenstein and Hawking on black hole thermodynamics, the entropic character of space-time has been linked to the very existence of black holes and to the notion of Bekenstein–Hawking entropy. Indeed, it is related as such to the area of their event horizons, a property expressed by the well-established Bekenstein–Hawking surface-area entropic relation. In subsequent literature, the Bekenstein–Hawking entropy has become a popular subject of investigation. This includes in particular the search for possible alternative routes to quantum gravity, to be typically intended as sort of microscopic descriptions of space-time in which classical GR should represent the macroscopic (or thermodynamic) continuum limit. According to this picture, the quantum description of the gravitational field achieved in this way should actually provide quantum corrections to the classical surface-area Bekenstein–Hawking relation.

Such developments are certainly interesting and worth serious consideration. The notion of quantum entropy, however, has a wider conceptual and mathematical extension, reaching beyond the concept of Bekenstein–Hawking entropy, or similar notions, which are associated with event horizons.

Indeed, as described in this paper, an alternate approach exists in which the notion of quantum entropy follows from first principles and a suitable formulation of quantum gravity itself. In particular, for this purpose, an approach based on the manifestly-covariant quantum gravity theory (CQG-theory) has been adopted. In such a case, as shown here, quantum entropy can be identified with the measure of ignorance on the corresponding quantum state, i.e., prescribed in terms of the quantum Boltzmann–Shannon entropy associated with the quantum probability density function (PDF) determined by the same CQG-theory.

The main results can be summarized as follows.

The first one concerns the non-unitary generalization of CQG-theory (Section 2) carried out in order to take into account the possible presence of an effective quantum sink, i.e., a graviton loss mechanism, which can occur sufficiently close to an event horizon (here for definiteness, this has been associated with the de Sitter space-time cosmological solution). For this purpose, based on a generalized Lagrangian path representation of the generally non-unitary quantum state, an explicit Gaussian solution has been determined for the corresponding quantum PDF (Section 3).

The second result refers to the explicit determination of the quantum BS entropy (i.e., identified with the so-called CQG-BS entropy), together with its entropy production rate and the corresponding suitably-normalized quantities. Both are achieved in the context of the non-unitary generalization of CQG-theory, a result that is significant in itself for its conceptual implications since it permits the extension of the theory to the event horizon (EH) surface and the surrounding EH-capture domain.

The third result concerns the analysis of the qualitative behavior of the CQG-BS entropy in different cases, including:

- *Case #1*: the entropic behavior occurring close to the EH, i.e., in the so-called EH-capture domain.
- *Case #2*: the entropic behavior in the external EH domain for a stationary background space-time.
- *Case #3*: the entropic asymptotic behavior in the limit s→+∞.

In all the cases indicated above, the normalized CQG-BS entropy has been shown to be monotonically increasing (*Case #1*), thus implying the validity of a strong H-theorem and exhibiting an irreversible proper-time behavior, or, respectively, to remain constant (*Cases #2 and #3*).

These results are useful to display novel features and physical characterizations of quantum gravity and its statistical properties achieved in the context of CQG-theory. In particular, the notion of quantum entropy adopted in this paper can be applied to generally non-stationary quantum states occurring in the presence of external (classical or quantum) sources. These outcomes are expected to support further studies on the statistical and thermodynamic properties of the gravitational field in the context of candidate quantum gravity theories and among alternative approaches to the issue available in the literature.

## Appendix A. Notations for the Classical and Quantum Hamiltonian Structures of GR

In this Appendix, following [1,2,3,42,43,44,45], we recall the relevant definitions involved in the classical and quantum Hamiltonian structures of GR appearing in the Hamilton–Jacobi quantization scheme (14)–(17). This is represented by a set $\left\{x_{R},H_{R}\right\}$, formed by an appropriate four-tensor canonical state $x_{R}\equiv \left(g_{\mu \nu },\pi^{\mu \nu }\right)$ and a corresponding four-scalar classical Hamiltonian density $H_{R}$. The latter is identified with the function:

(A1) $$H_{R}\equiv T_{R}+V,$$

where the effective kinetic and the normalized effective potential densities $T_{R}$ and *V* take the form (see [1,2]):

(A2) $$\left\{\begin{array}{c} T_{R}\equiv \frac{1}{2\alpha L}\pi_{\mu \nu }\pi^{\mu \nu },\\ V\left(g,\hat{g},r,s\right)\equiv V_{o}\left(g,\hat{g},r,s\right)+V_{F}\left(g,\hat{g},r,s\right). \end{array}\right.$$

Here, $V_{o}$ and $V_{F}$ represent the vacuum and external field contributions:

(A3) $$\begin{array}{c} V_{o}\equiv h\alpha L\left[g^{\mu \nu }{\hat{R}}_{\mu \nu }-2\Lambda \right],\\ V_{F}\left(g,\hat{g},r\right)\equiv hL_{F}\left(g,\hat{g},r\right), \end{array}$$

where ${\hat{R}}_{\mu \nu }\equiv R_{\mu \nu }\left(\hat{g}\right)$ and Λ identify respectively the background Ricci tensor and the cosmological constant, $L_{F}$ being associated with a possible non-vanishing stress-energy tensor. In addition, *h* is the variational weight-factor:

(A4) $$h\left(g,\hat{g}\left(r,s\right)\right)=2-\frac{1}{4}g^{\alpha \beta }g^{\mu \nu }{\hat{g}}_{\alpha \mu }\left(r,s\right){\hat{g}}_{\beta \nu }\left(r,s\right),$$

while *L* and α are constants, i.e., suitable four-scalars, both identified according to the treatment given in [2].

Second, let us recall the prescription of the total covariant *s*-derivative adopted in [3]. In Eulerian form, this is defined by:

(A5) $$\frac{d}{ds}={\left.\frac{d}{ds}\right|}_{s}+{\left.\frac{d}{ds}\right|}_{r},$$

where ${\left.\frac{d}{ds}\right|}_{s}\equiv t^{\alpha }\nabla_{\alpha }$ identifies the *directional covariant derivative*, with:

(A6) $$t^{\alpha }=\frac{dr^{\alpha }\left(s\right)}{ds}\equiv {\left.\frac{d}{ds}\right|}_{s}r^{\alpha }\left(s\right)$$

being the tangent to the geodetic curve $r\left(s\right)\equiv \left\{r^{\alpha }\left(s\right)\right\}$. In addition, ${\left.\frac{d}{ds}\right|}_{r}$ denotes the *covariant s*-*partial derivative.* When it operates on a four-scalar, this coincides with the ordinary partial derivative, so that ${\left.\frac{d}{ds}\right|}_{r}=\frac{\partial }{\partial s}$, and consequently, in this case:

(A7) $$\frac{d}{ds}={\left.\frac{d}{ds}\right|}_{s}+\frac{\partial }{\partial s}\equiv D_{s},$$

with $D_{s}$ to be referred to on the whole as the *convective derivative*.

Finally, let us recall the notations for the quantum variables and operators. Thus, denoting $\pi_{\mu \nu }^{\left(q\right)}\equiv g_{\mu \nu },$ $p^{\left(q\right)}\equiv -i\hbar \frac{\partial }{\partial g^{\mu \nu }}$ respectively the canonical generalized coordinates and conjugate quantum canonical momenta, the quantum Hamiltonian operator $H_{R}^{\left(q\right)}$ is taken in the form:

(A8) $$\begin{array}{ccc} H_{R}^{\left(q\right)} & \equiv & T_{R}^{\left(q\right)}\left(\pi ,\hat{g}\right)+V, \end{array}$$

(A9) $$\begin{array}{ccc} T_{R}^{\left(q\right)}\left(\pi ,\hat{g}\right) & = & \frac{1}{2\alpha L}\left(-i\hbar \frac{\partial }{\partial g^{\mu \nu }}\right)\left(-i\hbar \frac{\partial }{\partial g_{{}^{\mu \nu }}}\right), \end{array}$$

with $T_{R}^{\left(q\right)}\left(\pi ,\hat{g}\right)$ being the quantum effective kinetic energy operator and *V* being the effective potential prescribed according to Equation (A3).

## References

[1] Cremaschini C., Tessarotto M. (2017). Hamiltonian approach to GR—Part 1: Covariant theory of classical gravity. Eur. Phys. J. C.

[2] Cremaschini C., Tessarotto M. (2017). Hamiltonian approach to GR—Part 2: Covariant theory of quantum gravity. Eur. Phys. J. C.

[3] Cremaschini C., Tessarotto M. (2018). Space-time second-quantization effects and the quantum origin of cosmological constant in covariant quantum gravity. Symmetry.

[4] Boltzmann L. (1872). Weitere Studien über das Wärmegleichgewicht unter Gasmolekülen.

[5] Grad H. (1958). Thermodynamics of gases. Handb. Phys..

[6] Dunkel J., Trigger S.A. (2005). Time-dependent entropy of simple quantum model systems. Phys. Rev. A.

[7] Marchetti D.H.U., Wreszinski W.F. (2013). Asymptotic Time Decay in Quantum Physics.

[8] Shannon C.E. (1948). A Mathematical Theory of Communication. Bell Syst. Tech. J..

[9] Tessarotto M., Cremaschini C. (2014). The Master kinetic equation for the statistical treatment of the Boltzmann-Sinai classical dynamical system. Eur. Phys. J. Plus.

[10] Tessarotto M., Cremaschini C., Mond M., Asci C., Tironi A.S.G. (2018). On the Boltzmann-Grad Limit for Smooth Hard-Sphere Systems. Found. Phys..

[11] Tessarotto M., Cremaschini C. (2018). Macroscopic irreversibility and decay to kinetic equilibrium of the 1-body PDF for finite hard-sphere systems. Adv. Math. Phys..

[12] Han X., Wu B. (2015). Entropy for quantum pure states and quantum H theorem. Phys. Rev. E.

[13] Solano-Carrillo E., Millis A.J. (2016). Theory of entropy production in quantum many-body systems. Phys. Rev. B.

[14] Tessarotto M., Mond M., Batic D. (2016). Hamiltonian Structure of the Schrödinger Classical Dynamical System. Found. Phys..

[15] Tessarotto M., Cremaschini C. (2016). Generalized Lagrangian-path representation of non-relativistic quantum mechanics. Found. Phys..

[16] Jaynes E.T. (1957). Information Theory and Statistical Mechanics I. Phys. Rev..

[17] Jaynes E.T. (1957). Information Theory and Statistical Mechanics II. Phys. Rev..

[18] Mehta P., Andrei N. (2008). Nonequilibrium Quantum Impurities: From Entropy Production to Information Theory. Phys. Rev. Lett..

[19] Weilenmann M., Kraemer L., Faist P., Renner R. (2016). Axiomatic Relation between Thermodynamic and Information-Theoretic Entropies. Phys. Rev. Lett..

[20] Swendsen R.H. (2017). The definition of the thermodynamic entropy in statistical mechanics. Phys. A Stat. Mech. Its Appl..

[21] Ellerman D. (2018). Logical Entropy: Introduction to Classical and Quantum Logical Information Theory. Entropy.

[22] Bardeen J.M., Carter B., Hawking S.W. (1973). The four laws of black hole mechanics. Commun. Math. Phys..

[23] Bekenstein J.D. (1974). Generalized second law of thermodynamics in black-hole physics. Phys. Rev. D.

[24] Bekenstein J.D. (1973). Black Holes and Entropy. Phys. Rev. D.

[25] Hawking S.W. (1976). Black holes and thermodynamics. Phys. Rev. D.

[26] Tsallis C., Cirto L.J.L. (2013). Black hole thermodynamical entropy. Eur. Phys. J. C.

[27] Jacobson T. (1995). Thermodynamics of Spacetime: The Einstein Equation of State. Phys. Rev. Lett..

[28] Bhattacharya S., Shankaranarayanan S. (2015). How emergent is gravity?. Int. J. Mod. Phys. D.

[29] Padmanabhan T. (2015). Emergent gravity paradigm: Recent progress. Mod. Phys. Lett. A.

[30] Faizal M., Ashour A., Alcheikh M., Alasfar L., Mahroussah S.A.A. (2017). Quantum fluctuations from thermal fluctuations in Jacobson formalism. Eur. Phys. J. C.

[31] Susskind L., Uglum J. (1994). Black hole entropy in canonical quantum gravity and superstring theory. Phys. Rev. D.

[32] Saida H. (2011). Universal Property of Quantum Gravity implied by Uniqueness Theorem of Bekenstein-Hawking Entropy. Entropy.

[33] Zhang B. (2015). Entropy in the interior of a black hole and thermodynamics. Phys. Rev. D.

[34] Barbero G J.F., Villase nor E.J.S. (2009). On the computation of black hole entropy in loop quantum gravity. Class. Quantum Gravity.

[35] Nomura Y., Weinberg S.J. (2014). Black holes, entropies, and semiclassical spacetime in quantum gravity. J. High Energy Phys..

[36] Bodendorfer N., Neiman Y. (2014). Wald entropy formula and loop quantum gravity. Phys. Rev. D.

[37] Pranzetti D., Sahlmann H. (2015). Horizon entropy with loop quantum gravity methods. Phys. Lett. B.

[38] Blaschke M., Stuchlík Z., Blaschke F., Blaschke P. (2017). Classical corrections to black hole entropy in d dimensions: A rear window to quantum gravity?. Phys. Rev. D.

[39] Grüber D., Sahlmann H., Zilker T. (2018). Geometry and entanglement entropy of surfaces in loop quantum gravity. Phys. Rev. D.

[40] von Neumann J. (1955). Mathematical Foundations of Quantum Mechanics.

[41] Dirac P.A.M. (1936). The Principles of Quantum Mechanics.

[42] Cremaschini C., Tessarotto M. (2015). Synchronous Lagrangian variational principles in General Relativity. Eur. Phys. J. Plus.

[43] Cremaschini C., Tessarotto M. (2016). Manifest covariant Hamiltonian theory of General Relativity. Appl. Phys. Res..

[44] Cremaschini C., Tessarotto M. (2017). Quantum-wave equation and Heisenberg inequalities of covariant quantum gravity. Entropy.

[45] Tessarotto M., Cremaschini C. (2018). Generalized Lagrangian path approach to manifestly-covariant quantum gravity theory. Entropy.

[46] Messiah A. (1999). Quantum Mechanics.

[47] Einstein A. (2004). The Meaning of Relativity.

[48] Landau L.D., Lifschitz E.M. (1957). Field Theory, Theoretical Physics.

[49] Misner C.W., Thorne K.S., Wheeler J.A. (1973). Gravitation.

[50] Tessarotto M., Cremaschini C. (2016). Theory of Nonlocal Point Transformations in General Relativity. Adv. Math. Phys..

[51] Gaset J., Román-Roy N. (2018). Multisymplectic unified formalism for Einstein-Hilbert gravity. J. Math. Phys..

[52] Bohm D. (1952). Reply to a criticism of a causal re-interpretation of the quantum theory. Phys. Rev..

[53] Bohm D., Hiley B.J., Kaloyerou P.N. (1987). An ontological basis for the quantum theory. Phys. Rep..

[54] Grössing G. (2009). On the thermodynamic origin of the quantum potential. Phys. A Stat. Mech. Its Appl..

[55] Dennis G., de Gosson M.A., Hiley B.J. (2015). Bohm’s quantum potential as an internal energy. Phys. Lett. A.

[56] Struyve W., Valentini A. (2009). De Broglie-Bohmguidance Equations Arbitr. Hamiltonians. J. Phys. A Math. Theor..

